# Increased Diagnostic Yield of Capsule Endoscopy in Patients with Chronic Abdominal Pain

**DOI:** 10.1371/journal.pone.0087396

**Published:** 2014-01-31

**Authors:** Liping Yang, Yu Chen, Bingling Zhang, Chunxiao Chen, Min Yue, Juan Du, Chaohui Yu, Youming Li

**Affiliations:** Department of Gastroenterology, the First Affiliated Hospital, College of Medicine, Zhejiang University, Hangzhou, China; University of Modena & Reggio Emilia, Italy

## Abstract

**Background and Study Aims:**

Chronic abdominal pain is one of the most common chief complaints, but the underlying pathophysiology often remains unknown after routine clinical evaluation. Capsule endoscopy (CE) is a new technique for the visualization of the entire small bowel. The aim of this study was to evaluate the diagnostic efficacy of CE in patients with chronic abdominal pain of obscure origin.

**Patients and Methods:**

Two hundred forty three patients with chronic abdominal pain with no significant lesions were enrolled in this study. CE was performed in all patients.

**Results:**

A diagnosis was made in 23.0% of patients screened with CE. Of the 243 patients, 19 (7.8%) were diagnosed with Crohn's disease, 15 (6.2%) with enteritis, 11 (4.5%) with idiopathic intestinal lymphangiectasia, 5 (2.1%) with uncinariasis, and a number of other diagnoses including small bowel tumor, ascariasis, and anaphylactoid purpura. Five patients had abnormal transit time, and capsule retention occurred in two patients.

**Conclusions:**

In contrast to other previous studies, we found that CE is an effective diagnostic tool for patients with abdominal pain.

## Introduction

Chronic abdominal pain (CAP) that persists either continuously or intermittently for more than three months is one of the most common chief complaints in clinical medicine. The causes of abdominal pain are varied and complex, and include both functional and structural gastrointestinal abnormalities. Unfortunately, in many patients with CAP, the underlying cause of the disease remains unknown even after routine laboratory testing.

Capsule endoscopy (CE) is a new technology for painless endoscopic imaging of the entire small bowel [1], [2]. To date, several studies have demonstrated that CE is superior to barium follow-through, push enteroscopy, and computed tomography (CT) for the diagnosis of small bowel disease [3]–[5], and more specifically for the evaluation of patients with obscure gastrointestinal bleeding [6]–[10] and Crohn's disease [11]–[14]. However, there is a paucity of data regarding the diagnostic yield of CE in patients with CAP, and in previously published studies, CE was not informative in these patients [15], [16]. The primary objective of our study was to determine the diagnostic efficacy of CE in patients with CAP of obscure origin.

## Methods

### Ethics statement

After informed of the benefits and potential risks of capsule endoscopy examination, all the patients signed an informed consent form prior to their enrollment for capsule endoscopy examination. In addition, all the patients agreed that their clinical examination results could be used for non-commercial teaching or scientific research purposes and these verbal consents were recorded in the patient file. All the patient information was anonymized prior to analysis. The Ethical Review Committee (the First Affiliated Hospital, College of Medicine, Zhejiang University) approved this retrospective study and stated that “This study appears to be in accordance with the scientific principles generally accepted and to the ethical standards of research. The study was led in the respect of the Chinese law and regulation”.

### Patients and controls

Inpatients and outpatients who complained of either continuous or intermittent abdominal pain for at least three months at the First Affiliated Hospital, College of Medicine, Zhejiang University between January 2006 and December 2012 were screened for enrollment in this study. Patients were excluded if they presented with any of the following conditions: pregnancy, symptoms/signs of bowel obstruction, presence of implanted pacemaker, swallowing disorders, and any abdominal surgery except appendicectomy. Two hundred forty three patients (136 male and 107 female, with mean age of 44.1 years) were enrolled in this study.

Out of 243 patients, 112 patients complained of mid-epigastric pain, 37 of lower abdominal pain, and 94 of diffuse abdominal pain. All patients underwent routine clinical examinations and laboratory tests (including blood tests, urine tests, and stool examinations), ultrasound examinations or computed tomography (CT), esophagogastroduodenoscopy, and colonoscopy. We did not perform any barium studies as they are rarely used in our hospital. The studies did not reveal any obvious lesions that could contribute to clinical manifestations of disease.

### Capsule endoscopy

For CE, 144 patients were examined by OMOM CE system (JinShan Science & Technology Co, Ltd, ChongQing, China) and 99 patients were examined by Given PillCam SB Diagnostic System (Given Imaging Ltd., Yoqneam, Israel).

The patients were maintained on a liquid diet for one day and fasted for at least 10 hours prior to the examination. When the capsule was activated, the patient ingested it with one glass of water. Two hours after swallowing the capsule patients were allowed to drink water and in another two hours could have a light snack. The examination lasted approximately eight hours, and during this time the patient avoided any physical activity that involved sweating and abstained from bending. At the end of the procedure, the sensor belt and data recorder were removed by the physician.

### Statistical analysis

All the data recorded were analyzed by professional gastroenterologists.

## Results

Demographic data and clinical characteristics of the study population are listed in Table 1. Of the 243 patients evaluated, 56 patients were diagnosed with abnormalities in the small intestine (yield 23.0%). Forty four of these patients presented with periumbilial pain as the primary symptom (yield 78.6%). Specific CE findings included ulcers, erythema and edema, occupation, lymphangiectasia, diverticulum, fistula, uncinariasis, and ascariasis. The presumed diagnoses included Crohn's disease (19 patients), enteritis (15), idiopathic intestinal lymphangiectasia (11), uncinariasis (five), small bowel occupying lesion (three), ascariasis (two), and anaphylactoid purpura (one) (Table 2).

**Table 1:** Demographic data and clinical characteristics of the study population.

Characteristics	OMOM	PillCam SB	Total
No. of patients	144	99	243
Sex			
Male	75	61	136
Female	69	38	107
Median age (range), years	44.9(15–76)	44.0(9–79)	44.1(9–79)
Positive findings	32	24	56
Yield, %	22.2%	24.2%	23.0%

**Table 2:** CE findings in 243 patients with chronic abdominal pain.

	OMOM	PillCam SB	Total
No. of patients	144	99	243
Specific capsule endoscopy findings			
Ileal and jejuna ulcers	23	19	42
Ileal occupation	1	0	1
Jejunal occupation	0	1	1
Intestinal lymphandiectasia	9	2	11
Erythema and edema in jejuna and ileum	4	3	7
Ileal and jejuna polyps	1	0	1
Uncinariasis	5	0	5
Ascariasis	0	2	2
Diverticulum	1	0	1
Fistula	0	1	1
Follicular hyperplasia	1	2	3
Presumed diagnosis after capsule endoscopy			
Crohn's disease	9	10	19
Enteritis	8	7	15
Idiopathic intestinal lymphangiectasia	9	2	11
Uncinariasis	5	0	5
Ascariasis	0	2	2
Small bowel occupying lesion	1	2	3
Anaphylactoid purpura	0	1	1

**Figure 1:**
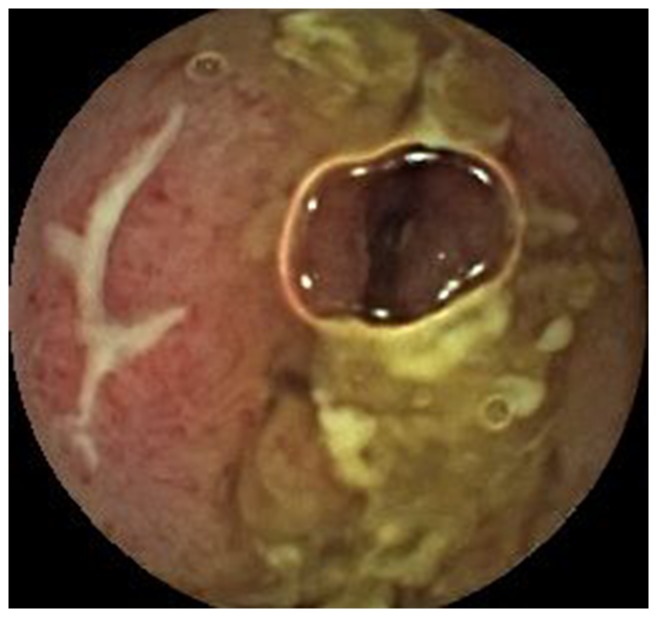
Ulcers and stenosis found by CE. Ulcers and stenosis in the upper part of the small intestine were found by CE in a 56-year-old woman with abdominal pain. The diagnosis of Crohn's disease was confirmed both surgically and pathologically.

**Figure 2:**
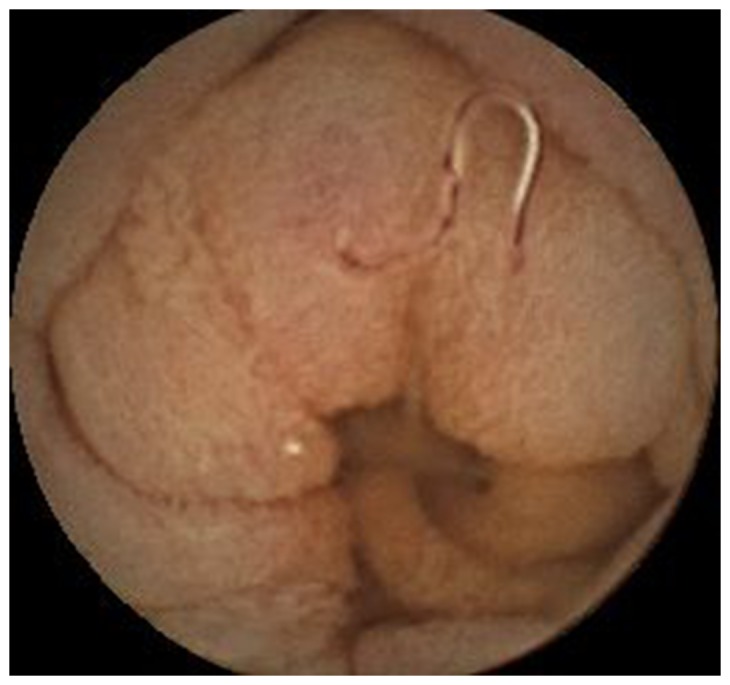
Hookworms identified by CE. Hookworms throughout the small bowel were identified by CE in a 42-year-old woman with lower abdominal pain for three years.

**Figure 3:**
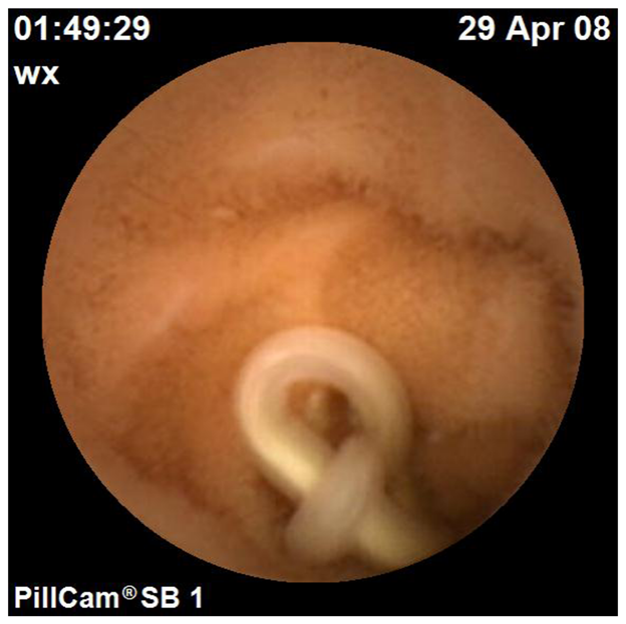
Roundworm detected by CE. Roundworm was detected by CE in the small bowel in a 49-year-old woman with periumbilial pain for more than one year.

**Figure 4:**
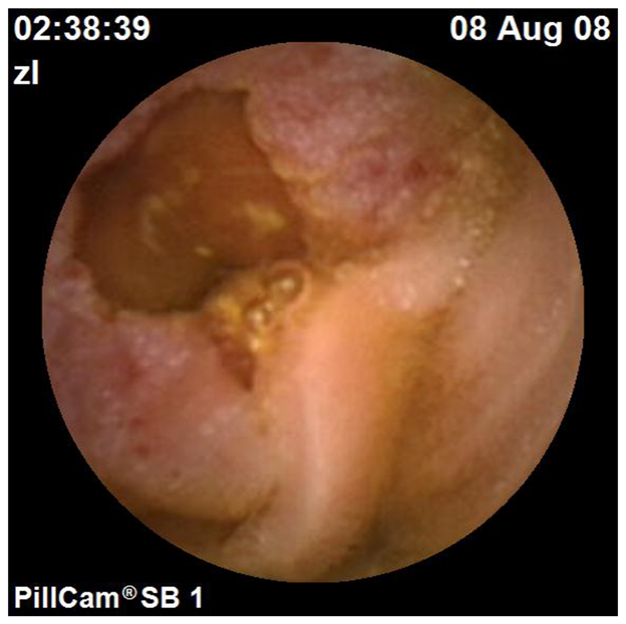
An occupying lesion identified by CE. An occupying lesion was identified by CE in the jejunum of a 52-year-old woman with abdominal pain for more than three months. With subsequent pathology evaluation, a diagnosis of adenocarcinoma was confirmed.

**Figure 5:**
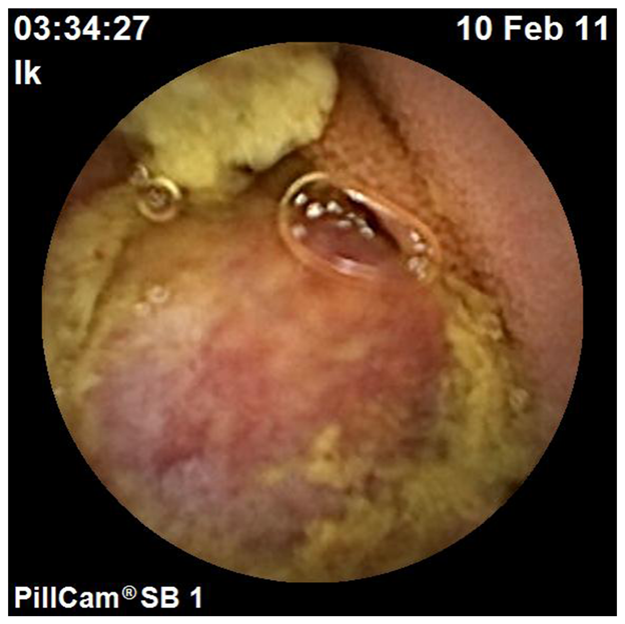
An eminence lesion identified by CE. An eminence lesion was identified by CE in the small bowel of a 30-year-old man with periumbilial pain. The lesion was surgically diagnosed as gastrointestinal stromal tumor.

**Figure 6:**
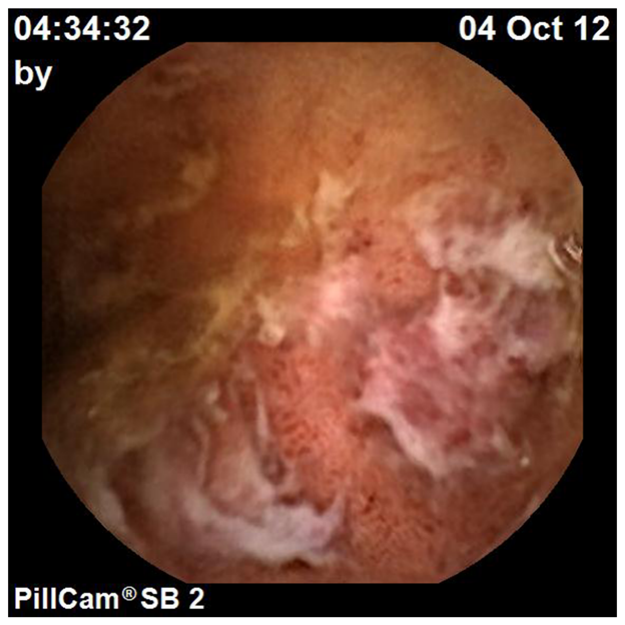
Multiple congestion and ulcers identified by CE. Multiple congestion and ulcers in the small bowel were identified by CE in a 17-year-old man with recurrent abdominal pain.

The diagnosis of Crohn's disease was based on the following criteria: ulcers, mucosal erosion, ileal ulcerated stenosis, and featureless small bowel [11], [17], [18]. CE detected roundworms (*Ascaris lumbricoides*) and hookworms (*ancylostome*) in patients with ascariasis and uncinariasis, which was previously undetected in the stool examination. The patient with occupation in the proximal jejunum was diagnosed with adenocarcinoma by pathological findings. The patient with small bowel occupying lesion was diagnosed surgically with gastrointestinal stromal tumor. The patient with anaphylactoid purpura had multiple congestion and ulcers in the small bowel (Figures 1, 2, 3, 4, 5, and 6).

Five patients exhibited abnormal transit time of the capsule. One had a prolonged transit time (1 hour 22 minutes to leave the stomach and 9 hours to reach the colon) and four had a rapid transit time (on average, 1 hour 15 minutes to reach the colon). Capsule retention occurred in two patients (0.82%), and localization was confirmed by X-ray KUB in the stomach and pelvic cavity.

## Discussion

For gastroenterologists, the small intestine is known as the “Dark Continent” because of the inherent difficultly to visualize this organ enteroscopically. In particular, small-bowel endoscopy is constrained by procedural discomfort and limits on the advancement of enteroscopes into the small bowel [1]. CE, which allows painless endoscopic imaging of the whole small bowel, has become a very important tool for the diagnosis of many small-bowel disorders [1], [19]. For example, gastrointestinal bleeding is the primary and most frequent indication for CE. As reported by various studies, the overall diagnostic yield for obscure gastrointestinal bleeding is in the range of 55%–70%, which is much higher than that for other diagnostic tests [5]–[10], [19]. CE was also efficacious for the diagnosis of small bowel lesions in Crohn's disease, suggesting it may be useful for early disease management [11]–[14], [19]. Moreover, CE can be used to diagnose patients with celiac disease and hereditary polyposis syndromes [20]–[22]. More recently, small bowel tumors have been detected with CE, where 6–9% of patients were diagnosed small bowel tumors [23]–[25]. Some studies revealed that CE could help to identify the intensity and extent of the gastrointestinal involvement in patients with anaphylactoid purpura [26]–[28].

In contrast to findings of other investigators, we demonstrated that CE increased diagnostic yield (23.0%) for patients with chronic abdominal pain of previously undetermined origin [15], [16]. We enrolled patients in our study who complained of continuous or intermittent abdominal pain for at least three months. Our findings made presumed diagnoses of Crohn's disease, enteritis, idiopathic intestinal lymphangiectasia, uncinariasis, small bowel occupying lesion, ascariasis, and anaphylactoid purpura in patients who presented with negative routine clinical examination. Therefore, our data suggest that CE is an effective diagnostic tool for the evaluation of patients with CAP.

The main advantage of our study over others is the enrollment of a large number of patients who had a previous negative clinical workup (including routine laboratory tests, esophagogastroduodenoscopy, and colonoscopy). There are some limitations, primarily the inherent defects of any retrospective study. Although data collection regarding details of every patient was a priority, tiny omissions were inevitable. Also, we used two kinds of CE, but we believe the intensive and elaborate analyses by our expert gastroenterologists make this potential limitation unlikely.

In conclusion, we found that visualization of the small bowel with CE is a diagnostically effective tool for patients with chronic abdominal pain of obscure origin. Our results have important implications for the diagnosis of multiple disorders of the small bowel and the development and refinement of appropriate treatment regimens clinically.

## References

[1] Iddan G, Meron G, Glukhovsky A, Swain P (2000) Wireless capsule endoscopy. Nature 405: 417. 10.1038/35013140 10839527

[2] Ginsberg GG, Barkun AN, Bosco JJ, Isenberg GA, Nguyen CC, et al (2002) Wireless capsule endoscopy: August 2002. Gastrointest Endosc 56(5): 621–4. 12397265 10.1016/s0016-5107(02)70106-5

[3] Appleyard M, Fireman Z, Glukhovsky A, Jacob H, Shreiver R, et al (2000) A randomized trial comparing wireless capsule endoscopy with push enteroscopy for the detection of small-bowel lesions. Gastroenterology 119(6): 1431–8. 11113063 10.1053/gast.2000.20844

[4] Costamagna G, Shah SK, Riccioni ME, Foschia F, Mutignani M, et al (2002) A prospective trial comparing radiographs and video capsule endoscopy for suspected small bowel disease. Gastroenterology 123(4): 999–1005. 12360460 10.1053/gast.2002.35988

[5] Hara AK, Leighton JA, Sharma VK, Fleischer DE (2004) Small bowel: preliminary comparison of capsule endoscopy with barium study and CT. Radiology 230: 260–265. 14617764 10.1148/radiol.2301021535

[6] Ell C, Remke S, May A, Helou L, Henrich R, et al (2002) The first prospective controlled trial comparing wireless capsule endoscopy with push enteroscopy in chronic gastrointestinal bleeding. Endoscopy 34(9): 685–9. 12195324 10.1055/s-2002-33446

[7] Lewis BS, Swain P (2002) Capsule endoscopy in the evaluation of patients with suspected small intestinal bleeding: Results of a pilot study. Gastrointest Endosc 56(3): 349–53. 12196771 10.1016/s0016-5107(02)70037-0

[8] Mylonaki M, Fritscher-Ravens A, Swain P (2003) Wireless capsule endoscopy: a comparison with push enteroscopy in patients with gastroscopy and colonoscopy negative gastrointestinal bleeding. Gut 52(8): 1122–6. 12865269 10.1136/gut.52.8.1122 PMC1773749

[9] Adler DG, Knipschield M, Gostout C (2004) A prospective comparison of capsule endoscopy and push enteroscopy in patients with GI bleeding of obscure origin. Gastrointest Endosc 59(4): 492–8. 15044884 10.1016/s0016-5107(03)02862-1

[10] Nakamura M, Niwa Y, Ohmiya N, Miyahara R, Ohashi A, et al (2006) Preliminary comparison of capsule endoscopy and double-ballon enteroscopy in patients with suspected small-bowel bleeding. Endoscopy 38(1): 59–66. 16429356 10.1055/s-2005-870446

[11] Fireman Z, Mahajna E, Broide E, Shapiro M, Fich L, et al (2003) Diagnosing small bowel Crohn's disease with wireless capsule endoscopy. Gut 52(3): 390–2. 12584221 10.1136/gut.52.3.390 PMC1773561

[12] Herrarías JM, Caunedo A, Rodríquez-Téllez M, Pellicer F, Herrerías JMJR (2003) Capsule endoscopy in patients with suspected Crohn's disease and negative endoscopy. Endoscopy 35(7): 564–8. 12822090 10.1055/s-2003-40241

[13] Chong AK, Taylor A, Miller A, Hennessy O, Connell W, et al (2005) Capsule endoscopy vs. push enteroscopy and enteroclysis in suspected small-bowel Crohn's disease. Gastrointest Endosc 61(2): 255–61. 15729235 10.1016/s0016-5107(04)02571-4

[14] Voderholzer WA, Beinhoelzl J, Rogalla P, Murrer S, Schachschal G, et al (2005) Small bowel involvement in Crohn's disease: a prospective comparison of wireless capsule endoscopy and computed tomography enteroclysis. Gut 54(3): 369–73. 15710985 10.1136/gut.2004.040055 PMC1774420

[15] Bardan E, Nadler M, Chowers Y, Fidder H, Bar-Meir S (2003) Capsule endoscopy for the evaluation of patients with chronic abdominal pain. Endoscopy 35(8): 688–9. 12929066 10.1055/s-2003-41520

[16] Fry LC, Carey EJ, Shiff AD, Heigh RI, Sharma VK, et al (2006) The yield of capsule endoscopy in patients with abdominal pain or diarrhea. Endoscopy 38(5): 498–502. 16767586 10.1055/s-2006-925340

[17] Dubcenco E, Jeejeebhoy KN, Petroniene R, Tang SJ, Zalev AH, et al (2005) Capsule endoscopy findings in patients with established and suspected small-bowel Crohn's disease: correlation with radiologic, endoscopic, and histologic findings. Gastrointest endosc 62: 538–544. 16185968 10.1016/j.gie.2005.06.026

[18] De Bona M, Bellumat A, Cian E, Valiante F, Moschini A, et al (2006) Capsule endoscopy findings in patients with suspected Crohn's disease and biochemical markers of inflammation. Dig Liver Dis 38(5): 331–5. 16569524 10.1016/j.dld.2006.02.004

[19] Rey JF, Ladas S, Alhassano A (2006) Kuznetsov K; ESGE Guidelines Committee (2006) European society of gastrointeastinal endoscopy (ESGE). Video capsule endoscopy: update to guidelines (May 2006). Endoscopy 38(10): 1047–53. 17058174 10.1055/s-2006-944874

[20] Petroniene R, Dubcenco E, Baker JP, Ottaway CA, Tang SJ, et al (2005) Given capsule endoscopy in celiac disease: evaluation of diagnostic accuracy and interobserver agreement. Am J Gastroenterol 100: 685–694. 15743369 10.1111/j.1572-0241.2005.41069.x

[21] Culliford A, Daly J, Diamond B, Rubin M, Green PH (2005) The value of wireless capsule endoscopy in patients with complicated celiac disease. Gastrointest Endosc 62: 55–60. 15990820 10.1016/s0016-5107(05)01566-x

[22] Mata A, Liac J, Castell A, Rovira JM, Pellisé M, et al (2005) A prospective trial comparing wireless capsule endoscopy and barium contrast series for small bowel surveillance in hereditary GI polyposis syndrome. Gastruintest Endosc 61: 721–725. 10.1016/s0016-5107(05)00289-0 15855978

[23] Cobrin GM, Pittman RH, Lewis BS (2004) Diagnosing small bowel tumors with capsule endoscopy. Gastrenterology 26 (Suppl (2)) AB1322. 10.1002/cncr.21975 16736516

[24] Bailey AA, Debinski HS, Appleyard MN, Remedios ML, Hooper JE, et al (2006) Diagnosis and outcome of small bowel tumors by capsule endoscopy: a three-center Australian experience. Am J Gastroenterol 101(10): 2237–43. 17032187 10.1111/j.1572-0241.2006.00749.x

[25] Cobrin GM, Pittman RH, Lewis BS (2006) Increased diagnostic yield of small bowel tumors with capsule endoscopy. Cancer 107(1): 22–7. 16736516 10.1002/cncr.21975

[26] Skogestad E (2005) Capsule endoscopy in Henoch-Schonlein purpura. Endoscopy 37(2): 189. 15692942 10.1055/s-2004-826188

[27] Preud'Homme DL, Michail S, Hodges C, Milliken T, Mezoff AG (2006) Use of wireless capsule endoscopy in the management of severe Henoch-Schonlein purpura. Pediatrics 118(3): e904–6. 16880250 10.1542/peds.2005-3111

[28] Ichikawa R, Hosoe N, Imaeda H, Takabayashi K, Bessho R, et al (2011) Evaluation of small-intestinal abnormalities in adult patients with Henoch-Schonlein purpura using video capsule. Endoscopy 43: E162–3. 21563063 10.1055/s-0030-1256266

